# Making sense of pediatric death: An exploratory qualitative study of emotion management strategies applied by the pediatric intensive care unit interprofessional team

**DOI:** 10.1177/26323524251393267

**Published:** 2025-11-12

**Authors:** Lisa Albrecht, Molly J. Ryan, Eva Ta, Jennifer R. Foster, Laura Buckley, Hillary Ferguson, Kathy Lyons, Amanda van Beinum, Karen Dryden-Palmer

**Affiliations:** 1 Children’s Hospital of Eastern Ontario Research Institute, Ottawa, ON, Canada; 2 Department of Pediatric Critical Care, IWK Health, Halifax, NS, Canada; 3 Department of Critical Care, Dalhousie University, Halifax, NS, Canada; 4 Department of Critical Care Medicine, The Hospital for Sick Children, Toronto, ON, Canada; 5 Lawrence Bloomberg Faculty of Nursing, University of Toronto, ON, Canada; 6 Department of Critical Care, Nova Scotia Health, Halifax, NS, Canada; 7 Department of Critical Care, Alberta Children’s Hospital, Calgary, AB, Canada; 8 Department of Sociology, York University, North York, ON, Canada; 9 Department of Critical Care, Child Health Evaluative Sciences, SickKids Research Institute, The Hospital for Sick Children, Toronto, ON, Canada

**Keywords:** emotional regulation, psychological distress, intensive care units (pediatric), health personnel, Canada, emotions, death, qualitative research

## Abstract

**Background:**

Caring for children at the end of life is a reality of practice in the pediatric intensive care unit (PICU). Learning how to make sense of death at work, and the emotions it entails, is necessary for all PICU professionals.

**Objective:**

To explore how PICU clinicians manage their emotions when encountering pediatric death at work.

**Design:**

Exploratory qualitative study grounded in interpretive phenomenology and the theoretical lens of emotional labor. We conducted one-time semi-structured interviews. Once transcribed, we inductively coded interview transcripts and subsequently generated themes through reflexive thematic analysis.

**Methods:**

Fifteen clinicians (*n* = 3 respiratory therapists; *n* = 3 physicians; *n* = 3 child life specialists; *n* = 2 nurses; *n* = 2 physiotherapists; *n* = 2 social workers) practicing in Canadian PICUs. The majority identified as women (*n* = 13). Four participants self-identified as Black, Indigenous, and/or a person of color.

**Results:**

We generated four themes that influenced how clinicians managed emotions related to death in PICU: (1) Figuring it out on the job; (2) Reframing and rationalizing death; (3) Managing emotions as quality end-of-life care; (4) Navigating organizational constraints. Although clinicians shared many strategies and resources for managing emotions, the ability to apply these strategies was impacted by systemic constraints (e.g., pace of work, understaffing) and unequal access across professions to unit-level resources.

**Conclusion:**

Navigating pediatric death in the workplace requires skilled emotional labor, and clinician access to appropriate support to manage its impacts, which varies by unit culture and profession. PICU leaders should facilitate unit- and individual-level supports that are inclusive of all team members.

## Plain language summary

Making sense of PICU death

(i) *What is already known about the topic?*

• End of life care in the pediatric intensive care unit (PICU) involves emotional labour, which comes with associated mental health risks.

• Despite the interprofessional nature of all PICU care, existing research on this phenomenon focuses primarily on the experience of nurses.

(ii) *What this paper adds*

• We interviewed interprofessional PICU staff to bring a holistic perspective on how the team manages emotions around death at work, underscoring uneven access to supports across professions.

• Emotion management strategies were shared across roles and career stages; these could be facilitated or impeded by individual and systemic factors.

(iii) *Implications for practice, theory or policy*

• Managing emotional labour associated with child death is exacerbated by constraints to accessing resources, particularly processing time immediately after the death and timely debriefs.

• PICU leaders and those in similar environments should consider the entire clinical team when designing mental health supports and advocate for systemic changes that support clinician wellbeing (e.g., efforts to improve retention).

## Introduction

Caring for children at the end of life is a reality of practice in the pediatric intensive care unit (PICU).^1–4^ There are known risks of exposure to child death for PICU clinicians^5–9^ that include post-traumatic stress disorders,^10^ feelings of grief,^11–14^ burnout,^15,16^ and moral distress.^17,18^ This has implications for individual clinicians’ long-term resilience and can impact retention in the clinical setting.^18–21^ PICU clinicians seek to make sense of pediatric death and the emotions these events evoke as a necessary element of their professional experience.

Clinicians working with dying children note that managing their emotions is a key part of providing end-of-life care, with a participant in a recent study stating that their “*biggest challenge [was] keeping [my] own emotions in check.*”^22^ This can be understood through the theoretical lens of emotional labor: “the invisible management of one’s emotions in order to perform one’s job.”^23^ Emotion management is the “work” of emotional labor – the specific strategies people use in order to meet tacit and/or explicit expectations of an acceptable emotional response for the situation.^24^ An ethnographic study of PICU nurses described a “crying etiquette”: “*a few tears are fine, but too many tears cross an invisible line*.”^11^ Like any type of labor, emotional labor can cause injury for the unequipped.^24,25^ Clinicians working with dying children have expressed feeling unprepared for the emotional labor their jobs required.^26–28^ Consequences of emotional labor can include feelings of alienation (e.g., distance from a sense of self and authentic emotional responses) and emotional exhaustion (e.g., numbness, fatigue, burnout).^24,25,29^

There is a need for a holistic understanding of emotional labor related to PICU end-of-life care. Although end-of-life care involves the PICU’s diverse, multi-disciplinary team,^30,31^ existing research on witnessing death in this setting primarily focuses on nurses’ experiences.^5,7,32^ Examining the entire PICU team can provide valuable context underlying the emotional experience of pediatric death and can identify opportunities to improve clinician education and training. We aimed to better understand how interprofessional PICU clinicians manage their emotions when encountering death at work.

## Methods

### Study design

We reported this study according to the COREQ reporting guidelines for qualitative research^33^ (Supplemental File 1).

This study was grounded in interpretive phenomenology, which involves interpreting the meaning of individuals’ lived experiences and supports our epistemological goals to produce insights on a complex and abstract phenomenon in an accessible and practical manner without sacrificing depth or nuance.^24,34^ Interpretive phenomenology acknowledges the researcher’s existing expertise^34^; this informed the composition of the research team and Steering Committee. We complemented this ontological grounding with the theoretical lens of emotional labor,^24^ which informed our interview guide design (e.g., how to ask clinicians about this often tacit phenomenon) and analytic interpretations (e.g., distinguishing between emotional labor and emotional impacts – in other words, paying attention to emotion as an expected or unavoidable part of the job and looking at emotional impacts of that labor, rather than assuming all emotional moments indicate emotional labor).

Reflexivity is an important concept in qualitative research; it involves questioning and critical reflection on researchers’ biases, experiences, and positionality in relation to the research at hand.^35^ See Table 1 for positionality considerations from the study leads.

**Table 1.** Study lead characteristics.

Study lead	Role(s) in data generation	Positionality considerations
Molly Ryan	Interviewer, involved in theme generation	• Early career cis-gender White woman living and working on unceded Indigenous land (Mi’kma’ki) in Canada • Formal educational training and previous professional experience in qualitative research • Working as PICU research coordinator at the time of data collection • Principal investigator for grant funding this study – this work builds on her undergraduate thesis work comparing the emotion management strategies used by doctors and funeral directors. Current work is informed by this experience (e.g., familiarity with literature, choice to apply Emotional Labor theory)
Eva Ta	Interviewer, primary coder, involved in theme generation	• Early career cis-gender East Asian woman living and working on unceded Indigenous land (Tkaronto) in Canada • Working as PICU research nurse coordinator at the time of data collection • Co-investigator for grant funding for this study • The current work is driven by the opportunity to merge experience as a frontline bedside nurse with prior graduate research experience in pediatric traumatic head injury
Lisa Albrecht	Primary coder, involved in theme generation	• Early career cis-gender White woman living and working on unceded Algonquin Anishinaabe territory • Working as PICU research coordinator at the time of data collection • Co-investigator for grant funding for this study • Work influenced by previous in-hospital work as phlebotomist, and external high school teaching experience
Karen Dryden-Palmer	Involved in theme generation	• Late-career cis-gender White woman living and working on unceded Indigenous land (Tkaronto) in Canada • Working as PICU clinical nurse specialist at the time of data collection and analysis • Senior qualitative methods expert • This work extends previous work in end-of-life care in pediatric critical illness and provider wellbeing in her Program of Well-being, Ethics and Resilience (POWER) research. Her research in this domain of pediatric critical care nursing is informed by 40 years of clinical nursing practice
Other considerations:		
• Participants knew that their interviewer was a research coordinator with qualitative expertise (M.R.), or a PICU research nurse coordinator who had recently received interview training (E.T.), respectively • The interviewer reflexively journaled pre- and post-interview to identify potential areas of bias and reflect on any unique aspects of the interview		

PICU: pediatric intensive care unit.

### Participant selection

This study involved English-speaking frontline clinicians practicing in a Canadian PICU who had encountered at least one death at work. We defined “clinician” inclusively as any individual providing direct care to children and/or their families. We circulated an online intake form through professional networks. This form included brief demographic screening questions to support purposeful sampling for maximum variation of experience and disciplinary background (Supplemental File 2). We aimed to recruit up to 15 participants as similar study designs with similar populations^11,13,26,27^ demonstrate that a sample as few as 6 to as many as 30 participants is sufficient to provide useful insights and identify areas for future in-depth work. Fifteen was large enough to include a range of professions, while small enough in scope to align with our exploratory aims.

### Data collection

A study team member (M.R., E.T.) obtained informed consent and conducted one-time, 30- to 60-minute, semi-structured interviews over video call or phone per participant preference. The semi-structured interview design ensured that interviews covered common questions about experiences with death at work while offering flexibility to follow-up on unanticipated topics that were meaningful to participants.^36^ The interview guide was refined by the Steering Committee and was piloted with a clinician working in an adjacent environment (cardiac PICU; Supplemental File 3).

Interviews were conducted at a pre-arranged day and time convenient for the participants. All interviews were audio-recorded and transcribed by a professional transcriptionist. Participants had the opportunity to review their de-identified transcripts prior to analysis. Interviewers completed field notes following each interview as part of an ongoing reflexive practice and to ensure that relevant non-verbal aspects of the data were recorded. Field notes were reviewed for context during the coding process but were not formally coded themselves.

### Data analysis

We followed the six-phase methodology for reflexive thematic analysis,^37^ with several adaptations to support multi-sited collaboration. Adaptations included two coders and a collaborative codebook to facilitate ongoing critical discussion and identify areas for probing in future interviews. Reflexive thematic analysis is well-suited to interpretive phenomenology; its flexibility and ability to support theory-informed analysis made it an ideal choice.^38^ These two approaches are also ontologically aligned, sharing the assumption that findings are generated by authors and the specific lenses they bring rather than assuming there is an objective truth that can be “found” or emerge neutrally from the data.^34,38^ Data were managed in Dedoose v4.12^39^ qualitative analysis software. All transcripts were first read in their entirety to gain a sense of the whole of the participant’s experiences. To develop an initial codebook, the first two transcripts were independently coded by the study leads (M.R., E.T., L.A., and K.D.P.). The remaining transcripts were inductively coded independently by two primary coders (E.T. and L.A.) who met regularly for collaborative critical discussions. Coding occurred while data collection was ongoing to facilitate interview guide refinement. Once the study leads collaboratively finalized the codebook (Supplemental File 4), we independently inductively generated themes by exploring key patterns within and between transcripts. Of the nine participants who indicated interest in reviewing preliminary themes at the time of the interview, three provided written feedback that informed the themes presented below.

## Results

**Figure 1.**
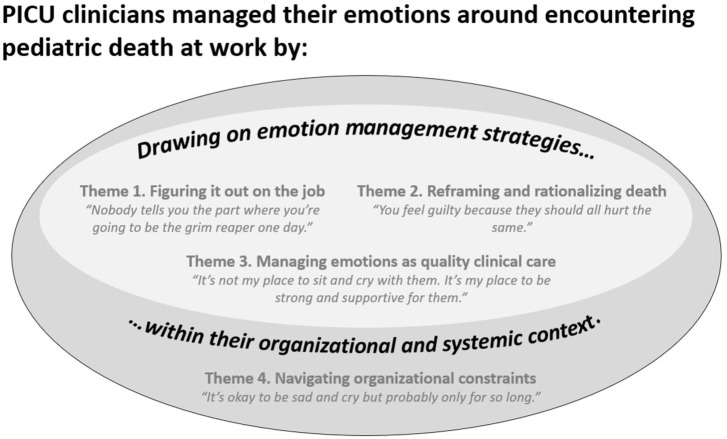
Overview of emotion management strategies used by PICU clinicians when encountering death. PICU: pediatric intensive care unit.

Fifty-seven clinicians completed the study intake form between October 2022 and March 2023. We contacted 18 participants; 2 declined, 1 was ineligible (adult ICU clinician). Between January 2023 and July 2023, we interviewed 15 clinicians (*n* = 6 different professions). See Table 2 for demographic details. Through reflexive thematic analysis, we generated four themes that reflected how PICU clinicians managed their emotions when encountering death at work (Figure 1).

**Table 2.** Demographics of study participants.

Participants (*N* = 15)	*n* (%)
Gender	
Man	2 (13)
Woman	13 (87)
Self-identifies as Black, Indigenous, and/or a person of color	
Yes	4 (27)
No	11 (73)
Profession	
Child life specialist	3 (20)
Nurse	2 (13)
Physician	3 (20)
Physiotherapist	2 (13)
Respiratory therapist	3 (20)
Social worker	2 (13)
Province	
British Columbia	2 (13)
Alberta	3 (20)
Ontario	5 (33)
Quebec	4 (27)
Nova Scotia	1 (7)
Years worked in PICU	
1–4	6 (40)
5–9	3 (20)
10–15	1 (7)
>15	5 (33)
Years worked in profession	
1–4	3 (20)
5–9	2 (13)
10–15	3 (20)
>15	7 (47)

PICU: pediatric intensive care unit.

***Theme 1: Figuring it out on the job*:** “*Nobody tells you the part where you’re going to be the grim reaper one day*.”

Most participants felt unprepared for the emotional labor that came with end-of-life care, which they learned how to navigate on the job: “*they don’t teach this in school. They teach you what to do, but they don’t teach you the emotional aspect of it, how you would feel*” (Respiratory therapist, practicing 15+ years). Clinicians across professions shared that formal training on death focused on its clinical aspects. Some participants felt this undermined their preparedness for the potential emotional impacts: “*There’s so much taboo around death that it made us much less prepared and much less informed of what is available to help us go through this. It’s almost like it undermined the impact it could have on us*” (Nurse practicing 1–4 years). PICU clinicians found that death was sometimes a “taken for granted” aspect of their jobs, with an unspoken expectation to “push through” or “figure it out” on their own: “*I feel there’s a huge lack of support for ourselves, the medical professionals. And we’re just expected to be superheroes and just keep going*” (Respiratory therapist, practicing 10–15 years). A culture of “pushing through” encouraged compartmentalization and obscured the need to learn strategies for handling death at work. A physiotherapist likened her experience of compartmentalization to forcing shoeboxes to fit into a full closet; she further questioned: “*What is processing? And it could be different for everybody. But what are the tools and steps?. . . It seems to be some sort of secret that nobody’s sharing*” (Physiotherapist, practicing 10–15 years). As most of her physiotherapy peers do not encounter pediatric death at work, this quote also reflected her sense of isolation and exclusion from informal means of learning how to manage death at work.

Some of the most effective resources for learning emotion management strategies and processing death at work were supportive relationships with colleagues. Many participants shared the importance of debriefing with colleagues: “*it’s helpful to like echo the sadness of it, and then you don’t feel so isolated in your thoughts of what just happened*” (Nurse, practicing 5–9 years). Late-career clinicians described mentoring and teaching junior staff about strategies for coping with the emotional aspects of death as part of work. However, this meant that sometimes these participants lacked support for themselves: “*But if you’re really struggling, it’s sort of like I would say [you need] an RT [respiratory therapist]. But you can’t have an RT that’s junior to you, that’s never experienced anything in their life, and see their life as, you know, get on and deal with it*” (Respiratory therapist, practicing 15+ years).

***Theme 2: Reframing and rationalizing death*:** “*You feel guilty because they should all hurt the same*.”

When reflecting on how they manage their emotions, interviewees spoke to the ways they made sense of death itself. For instance, spiritual beliefs helped reframe death for some clinicians: “*I tap into my faith and being grateful. I just thank God for giving me the opportunity to be part of this family’s experience, and for Him to give me what they need to guide me through that process*” (Child life specialist, practicing 15+ years). Several participants perceived an expectation that “planned” deaths would be easier than others:

Maybe there’s like an expectation that if it was planned, it should be easier somehow. And for me personally, it does feel a little bit easier. But maybe it only feels easier in the moment and not later, when you’re thinking about it later. So I don’t know. I don’t know if there’s an expectation that it should be better in some ways or not. I think maybe I have that expectation of myself, if I can see it coming and prepare for it mentally then it’s a little bit better. But it still never feels good. It still feels shocking, just to different degrees. (Nurse, practicing 5–9 years)

Similarly, a respiratory therapist described how “expected” deaths provided more time to rationalize death as the best outcome:

The more that you've had a chance to mentally prepare, and the more that it’s anticipated, then the easier I find it. . . you’ve sort of had an opportunity to process your feelings along the way. . . Generally speaking, these are situations where you’ve already kind of emotionally rationalized yourself that this is for the best, and why this is for the best. You’ve kind of made sense of it already. (Respiratory therapist, practicing 15+ years)

Many participants invoked the concept of a “good” or “bad” PICU death, acknowledging that some deaths require more emotional labor through mental reframing and rationalization than others. Knowing the patient and family well, including their vision of a good death, made it easier for clinicians to feel confident that they had successfully facilitated it:

I had a young lady, she actually ended up passing in the adult ICU but I knew her for her whole 24 years of life [. . .] And I remember asking her, I said, “What do you need in these last couple of days? Tell me what you need.” And she said, “I need one more sunrise” [. . .] I should have been so sad, but I felt so blessed to be part of that experience. (Child life specialist, practicing 15+ years)

Participants also noted that strong relationships, such as those built over years with children whose deaths were anticipated, could make coping with their loss more difficult:

I want to know this child, I want to know this family. I want to know everything about them while they’re here, and treat them as a package. And then when you get to know them, then also, too, death and dying is a little bit harder, harder on the soul. (Respiratory therapist, practicing 15+ years)

The framing of “good” or “bad” was influenced by contextual factors of the death itself (e.g., unexpected death, family reactions to death, death perceived as preventable or unjust), and by their professional values (e.g., privilege to facilitate death with dignity, family-centered care) and personal values (e.g., spiritual beliefs, personal experiences with death).

Participants consistently highlighted how no two therapeutic relationships are alike:

So, for sure, I would say the relationship that I have with the family definitely influences how I experience the death of the child. Though of course in all cases, like my role would remain the same. But for sure from a personal perspective, it’s different, you know, depending on the relationship with the family. (Social worker, practicing 15+ years)

Despite this, some described a tacit belief that “*all deaths [in PICU] should hurt the same*,” suggesting that the goal of reframing and rationalization is to present a uniform emotional response and level of emotional intimacy. Clinicians experienced tension between the personal impact of certain patient deaths and this perceived expectation of emotionally equal relationships with all patients and families.

***Theme 3: Managing emotions as part of quality clinical care*:** “*It’s not my place to sit and cry with them. It’s my place to be strong and supportive for them*.”

Managing emotional responses to death at work intersected with participants’ professional identity and their perception of doing a “good job.” Knowing they had done the “best job” clinically helped participants rationalize and process the experience:

Not that, you know, it’s still fair for that to happen. But it helps me process the emotions a little bit differently. That, okay, this is why it’s happening, and this is what we’ve done to help them. Like in terms of we’re doing everything we can to make this as best as possible of an experience. (Nurse, practicing 5–9 years)

Doing a “good job” often meant putting the family first. In practice, this looked like intentionally compartmentalizing emotions (e.g., focusing on the family, suppressing own emotions), medicalizing death (e.g., focusing on clinical aspects within the clinician’s control), and establishing mental boundaries (e.g., “*this is their loss, not yours*”). These norms were exemplified by a child life specialist:

They’re not our families. This is their journey. We can, as I said before, walk alongside them, but we’re not living it. Like they’re living it. So it’s not my place to sit and cry with them. It’s my place to be supportive and strong for them. I’ve had moments of tears, for sure. Even in the hospital, just not in their room with them. I’m human. So like there’s moments where like my eyes will tear up. But it’s important to keep it together. (Child life specialist, practicing 15+ years)

Centering the family and not acknowledging one’s own experience in the moment was framed as helpful by most interviewees: “*knowing I’ve done my job and I’ve [done] it well gives me peace, because I know I can’t change the rest*” (Social worker, practicing 1–4 years). When clinicians were prevented from doing what they perceived as the best job possible, they experienced guilt and had difficulty processing the experience:

The biggest and most frustrating deaths that have led to the greatest burden on individuals tend to be those where there may have been some modifiable issue, not necessarily a decision made by a clinician, but in the system they’re in. And that’s where that frustration emerges, and that’s where that guilt emerges, and so on. (Physician, practicing 10–15 years)

Clinicians navigated sharing the “right” amount of emotion with families. A broader range of emotional expression was acceptable with colleagues or at home, out of sight of patients and families. Across several professions doing a good job meant providing emotional support while controlling their own emotional expression: “*I can’t get like super emotionally and personally, you know, affected and like be, you know, a mess because then I’m not going to be helpful to the family*” (Social worker, practicing 15+ years). Participants noted that displaying some emotions to families was okay, and sometimes expected, but required a skillful navigation to ensure they did not “take away” from the family:

Like you can have emotions, and you can be sad with the family. And I think that might even be helpful for a family to see that, you know, like it’s really, really sad. But not to the point where you’re taking away from them. Or like I would like never want a family to like have to comfort a team member because they’re so sad and they’re crying. Like it’s not about us. (Social worker, practicing 1–4 years)

The effort to displace one’s emotions by focusing on the family’s needs sometimes caused “emotional whiplash,” as illustrated by a child life specialist:

I remember one year I had end of life, and it was the week of Christmas. And I was with the family, and I was doing the lock of hair and the handprint. And then I had to go upstairs and do the Christmas party, and help Santa hand out presents. And it was just the emotional rollercoaster of that day. (Child life specialist, practicing 5–9 years)

Many clinicians viewed an ideal unit culture as one flexible to each clinician’s emotions:

Every single experience is unique and is meaningful as much for the parent, the child and the healthcare worker who’s kind of closest to that. . .So I think that that’s a balance that’s very hard to maintain, but that’s really established on our unit. . .I’m very grateful for that because I’ll never feel like the emotions that I have towards accompanying a family with their child’s death, I never feel like those emotions will ever not be valid and not be heard. (Nurse, practicing 1–4 years)

Several participants shared that norms around appropriate emotional expression have shifted:

I think over the years we have gone through two extremes. One extreme in the beginning was you should not demonstrate any emotion at the bedside. It is not professional. You’re crossing the boundaries. . .Then we kind of went to the other extreme, to say, you know, you can bawl at the bedside, and that’s very important for the family to know that you’re feeling their child’s death. Well, then sometimes that could cause trouble. . .problems because you’ll be emotionally so involved that you kind of lose your perspective . . . I think there’s a middle ground that should be allowed and should be part of what you are as a professional. And my expectation, and I see this now clearly, is that shedding some tears at the bedside when a child dies is perfectly acceptable. (Physician, practicing 15+ years)

A norm that encourages more emotional involvement could inadvertently put pressure on clinicians to display more emotions than they feel in order to do a “good job.” In fact, an early career physician shared that they have occasionally questioned if they were feeling “enough”: “*I remember my staff was crying, and then they looked at me, and I was not crying. And I was kind of like, Oh, should I be? And it’s funny, I’ve never felt that in the opposite way. Like I’ve never felt like I shouldn’t be crying when I was*” (Physician, practicing 1–4 years).

***Theme 4: Navigating organizational constraints*:** “*It’s okay to be sad and cry but probably only for so long*.”

Clinicians encountered organizational constraints that limited their ability to apply their preferred emotion management strategies: “*No matter which site, which hospital you work for. . .everyone is over-worked. There’s not enough resources, and we don’t have time to process anything*” (Respiratory therapist, practicing 10–15 years). The pace of work, exacerbated by under-staffing, led some to draw on compartmentalization and other avoidant emotion management strategies:

Partially maybe it’s deliberate – to avoid those feelings, acknowledging that it’s still a work day and there’s still other decisions to make. And there needs to be some sort of ongoing process for decision-making that’s not emotionally burdened. And so I tend to defer that grief until later. And sometimes it’ll, like, jump out in unexpected ways when you’re thinking about something else entirely. (Physician, practicing 10–15 years)

Many participants described how these suppressed emotions resurfaced:

And it’s like I don’t after a death sit down and deal with my emotions because often you can’t. You don’t have time. And it’s when it creeps up after in the locker room, and you’re crying, you need to get it out. You can’t suppress it. You’re crying all the way home in the car. Get it out. (Respiratory therapist, practicing 15+ years)

In contrast, one of the most effective emotion management strategies clinicians mentioned was being able to take a break following a death and/or having sufficient mental and physical space to process it. For example, ending their shift following a death:

There was really no expectation from my nurse in charge or from my team for me to continue working that day. And I think that’s something that should be a rule in every single healthcare facility. And I think that if you have to go through a traumatic event with a family, you should be allowed to do whatever you want to make yourself. . .to make the process of going through it easier. (Nurse, practicing 1–4 years)

The ability to take time or space post-death was rare for virtually all interviewees:

They would tell you that it’s okay to be however you’re going to be. . .But practically, no. Practically there’s one of me, and I’m running an ICU. . .That’s the part that’s hard, the part where the pace of the work does not allow for any buffer space. (Respiratory therapist, practicing 15+ years)

Participants sought systemic changes that addressed retention and short-staffing. There was also interest in further training (e.g., simulations) and improved interprofessional supports at the unit level as formal debriefs tended to include only physicians and nurses: “*I wish that we would actually have someone who would actually talk to us. . .Because we’re not part of the meetings when it comes to end-of-life care. . .We’re not there. But we’re doing everything to end the child’s life*” (Respiratory therapist, practicing 10–15 years). Further, the onus to “handle it” was often put back on the individual clinicians, rather than on hospital leadership to implement systems-level supports: “*Hospitals and institutions are blaming us that we don’t take care of ourselves. . ..it’s all upon us to respond to the situations of conflict, to the death of the children*” (Physician, practicing 15+ years). Some clinicians navigated the reality of emotional labor in PICU by considering leaving, with a nurse sharing that they could “*see the attractiveness of a normal job where there’s less emotional toll. . .better supporting staff is important for retention and to help address the emotional aspect of the job*” (Nurse, practicing 5–9 years). One participant briefly left the ICU setting:

I was just like “I’m done with this. . .I don’t want to have to deal with this anymore.” So I had left the ICU setting for about a year and I went into private. I wanted to completely disconnect, essentially. And I didn’t realize the amount of burden that I was carrying emotionally. And it was all because I never processed it. (Respiratory therapist, practicing 10–15 years)

This highlights a need to minimize organizational constraints that can exacerbate negative effects of this labor.

## Discussion

### Main findings of the study

Emotional labor is an important aspect of end-of-life care for all members of the PICU interprofessional team. Yet, managing emotions around death at work is an often underrecognized skill. For clinicians in this study, managing emotions meant needing to “figure it out” on the job, using strategies like reframing and rationalizing death, seeing emotion management as part of quality end-of-life care, and navigating organizational constraints. The lack of preparation received for the emotional nature of PICU work reflects previous studies.^26,27,40^ Emotional labor has been reported as systemically undervalued and invisible, with organizational interventions frequently shifting responsibility back on individuals to navigate system-level barriers.^41^ Barriers include time constraints, short-staffing, and lack of prioritization for clinician needs.^7,27,42^ Not having space to process emotions on the job^42,43^ can lead clinicians to rely on emotion management strategies like compartmentalization or avoidance, which exacerbates their risk of negative outcomes like burnout.^24,44^

### What this study adds?

Doing a “good job” at end-of-life care for clinicians in this study required highly-skilled emotional labor – the ability to demonstrate compassion and composure at the right times and with the right intensity. Framing emotional labor as a necessary skill is an example of “cognitive restructuring” – a recognized strategy for coping with the negative effects of emotional labor by reframing the experience positively.^44^ Further, managing emotions in front of families is not a self-imposed expectation; it aligns with and is actively reinforced by parental expectations of what quality PICU end-of-life care looks like. For example, an Australian study regarding bereaved parents’ judgments of the quality of PICU clinician’s service, parents described “*very good*” clinicians as those who “*displayed an emotional response to the child’s death*” and “*fantastic*” clinicians not only demonstrated compassion through appropriate emotional displays, they also were skilled at remaining calm during stressful events.^45^ This phenomenon was summed up succinctly by a participant in another study of bereaved parents’ impressions of PICU clinicians: “*I think that separates the good nurse from the bad nurse and the good doctor from the bad doctor – those that can cry with you, those that feel for you – but they still have their head on straight*.”^46^ Given the importance both clinicians and families place upon this emotion work, it deserves space in curricula and PICU onboarding.

Relationship-building with patients and families was a key phenomenon impacting the emotional experience of death at work for PICU clinicians. A recent qualitative study with American physicians also highlighted the importance of relationship-building in pediatric end-of-life care, with a physician sharing that forging connections was integral to “*being the kind of physician he wanted to be for them in the long run*.”^27^ Most participants in our study viewed emotional attachment as necessary to provide the “best” version of end-of-life care, and emotional detachment as a reluctant tool and risk to be managed (both for personal emotional injury and as something that patients and their families associate with worse care), rather than an inevitable aspect of the emotional labor required. This contrasts a study with adult ICU nurses that concluded “*a degree of emotional detachment [was] necessary in order to work in a critical care setting*.”^21^ Furthermore, PICU clinicians described the nuances of how a long relationship with patients can make the death easier (e.g., knowing the family and patient’s wishes and being able to fulfill them) and more difficult (e.g., death of someone they knew well and cared for). This ambivalence aligns with findings from a recent study^9^ on PICU clinician wellbeing that pushed against the idea of a continuum neatly separating risks from protective factors. The factors affecting well-being that they identified were not mutually exclusive but rather coexisted, much like the way in which relationship-building was not exclusively “positive” or “negative” for clinicians in our study.

Given the significance participants placed upon support from colleagues and involvement in team-level resources such as debriefs, the uneven availability and access across professions and career stages to these supports was concerning. Positive peer relationships help normalize emotions, validate experiences, and contribute to their long-term retention in PICU.^9^ Relying on more individualized emotion management approaches, like compartmentalization, whether intentional or due to contextual constraints, has been acknowledged as a potentially helpful strategy or could contribute to the experience of burnout.^24,47^ It may be that the individual’s motivation for compartmentalizing their experience and how they utilize that strategy is important in terms of both immediate and cumulative impacts.^48^ Certainly, burnout is a known risk in PICU providers. A recent systematic review of PICU physicians and nurses around the world found that 8 in 10 reported moderate or high levels of burnout.^49^ Similarly, one third of Canadian critical care physicians in a 2021 survey said moral distress was the reason they had considered leaving or previously left a position.^50^ Our findings reinforce the need to move away from seeing PICU’s emotional demands and systemic outcomes like burnout as individual issues to be managed privately or by trial and error, and instead shift the focus to what structural and systemic changes may better support clinicians moving forward.^27^

PICU leaders can promote a supportive environment for staff by prioritizing both proactive (e.g., dedicated onboarding regarding emotional impacts, simulations) and reactive institutional supports (e.g., refining team debriefing practice following death, access to counseling services) that are designed with the entire PICU team in mind.^18^ Similar supports were called for in a recent study of emotion management among Spanish palliative care nurses.^51^ Extra attention should be paid to groups who may have fewer intra-professional supports in the PICU environment (e.g., child life specialists, social workers, physiotherapists), while recognizing that all team members risk feeling isolated and could benefit from guidance on the emotion work of PICU care.^27^ Without comprehensive supports that acknowledge the emotional labor of all PICU team members, Canadian PICUs risk losing skilled clinicians to “*normal job[s]*” without these emotional demands.

### Strengths and limitations

This study contributes a holistic view of emotional labor in PICUs across Canada by recruiting a diverse sample of clinicians, rather than focusing on a single profession or hospital. The support of a national critical care research organization increased our reach. The study was strengthened by ongoing guidance from our Steering Committee, which included PICU clinicians (physician, nurse practitioner, social worker), qualitative methodological experts, and experienced ICU end-of-life researchers. We supported transparency in the data generation process by sending preliminary themes to participants for review and presenting emergent findings to critical care clinicians for feedback. This study has several limitations. Recruitment took place after the COVID-19-related clinician exodus from hospital settings,^52–55^ which may have excluded those most affected by the emotional labor in PICU. Furthermore, these findings may overrepresent experiences of staff who are reflective or distressed compared to staff with more neutral experiences and/or those who do not see a meaningful link between their emotional experience and the workplace. While these findings reflect a shared experience for interviewees across Canada, they do not offer a detailed case study of the norms and nuances around emotional labor within each hospital or profession. Although we aimed to include multiple professions, some key members of the PICU team were not represented. In addition to professions missing in our study (e.g., pharmacists, spiritual care professionals), future research would benefit from focusing on members of the PICU team who continue to be underrepresented in the literature (e.g., respiratory therapists, child life specialists). Lastly, while all 19 Canadian PICUs are located in urban areas and Canadian pediatric critical care capacity is similar to that of other high-income countries,^56^ staffing ratios, populations served, and the unique challenges associated with these cannot necessarily be translated to rural or lower-resourced areas. Nevertheless, comparable international studies do show similar findings. For instance, a 2025 study^51^ of emotion management among Spanish palliative care nurses reports nearly identical barriers to managing work experiences of death (staffing post-COVID, emotion management, lack of preparation), coping facilitators (relationships, personal experience, training), and desire for additional resources (emotion management training, psychosocial resources, and management intervention).

## Conclusion

Encountering death on the job is emotionally demanding, requiring skillful navigation of norms and expectations governing appropriate emotional responses in the moment, and meaning-making over time. This work has several practical implications for institutions and leaders to consider. PICU leaders should facilitate unit- and individual-level institutional supports that acknowledge situational and profession-specific factors inclusive of the entire care team. Emotional labor should be considered in clinical preparations for practice, including onboarding and debriefing considerations, and continuing education across career stages. Further exploration of mentorship, unit/organizational culture, and interdisciplinary components of this phenomenon is needed. Focused attention on mitigating organization-level barriers and systemic constraints on application of emotion management strategies is recommended.

## Supplemental Material

sj-docx-1-pcr-10.1177_26323524251393267 – Supplemental material for Making sense of pediatric death: An exploratory qualitative study of emotion management strategies applied by the pediatric intensive care unit interprofessional team Supplemental material, sj-docx-1-pcr-10.1177_26323524251393267 for Making sense of pediatric death: An exploratory qualitative study of emotion management strategies applied by the pediatric intensive care unit interprofessional team by Lisa Albrecht, Molly J. Ryan, Eva Ta, Jennifer R. Foster, Laura Buckley, Hillary Ferguson, Kathy Lyons, Amanda van Beinum and Karen Dryden-Palmer in Palliative Care and Social Practice

sj-docx-2-pcr-10.1177_26323524251393267 – Supplemental material for Making sense of pediatric death: An exploratory qualitative study of emotion management strategies applied by the pediatric intensive care unit interprofessional team Supplemental material, sj-docx-2-pcr-10.1177_26323524251393267 for Making sense of pediatric death: An exploratory qualitative study of emotion management strategies applied by the pediatric intensive care unit interprofessional team by Lisa Albrecht, Molly J. Ryan, Eva Ta, Jennifer R. Foster, Laura Buckley, Hillary Ferguson, Kathy Lyons, Amanda van Beinum and Karen Dryden-Palmer in Palliative Care and Social Practice

sj-docx-3-pcr-10.1177_26323524251393267 – Supplemental material for Making sense of pediatric death: An exploratory qualitative study of emotion management strategies applied by the pediatric intensive care unit interprofessional team Supplemental material, sj-docx-3-pcr-10.1177_26323524251393267 for Making sense of pediatric death: An exploratory qualitative study of emotion management strategies applied by the pediatric intensive care unit interprofessional team by Lisa Albrecht, Molly J. Ryan, Eva Ta, Jennifer R. Foster, Laura Buckley, Hillary Ferguson, Kathy Lyons, Amanda van Beinum and Karen Dryden-Palmer in Palliative Care and Social Practice

sj-docx-4-pcr-10.1177_26323524251393267 – Supplemental material for Making sense of pediatric death: An exploratory qualitative study of emotion management strategies applied by the pediatric intensive care unit interprofessional team Supplemental material, sj-docx-4-pcr-10.1177_26323524251393267 for Making sense of pediatric death: An exploratory qualitative study of emotion management strategies applied by the pediatric intensive care unit interprofessional team by Lisa Albrecht, Molly J. Ryan, Eva Ta, Jennifer R. Foster, Laura Buckley, Hillary Ferguson, Kathy Lyons, Amanda van Beinum and Karen Dryden-Palmer in Palliative Care and Social Practice
