# Supplementary material for: Making sense of pediatric death: An exploratory qualitative study of emotion management strategies applied by the pediatric intensive care unit interprofessional team
Source: Palliat Care Soc Pract. 2025 Nov 12;19:26323524251393267. doi: 10.1177/26323524251393267 (PMC12612547; doi:10.1177/26323524251393267)
Supplement: sj-docx-1-pcr-10.1177_26323524251393267 – Supplemental material for Making sense of pediatric death: An exploratory qualitative study of emotion management strategies applied by the pediatric intensive care unit interprofessional team [file sj-docx-1-pcr-10.1177_26323524251393267.docx]

**Appendix A:** Criteria for reporting qualitative studies (COREQ): 32-item checklist*

| **Checklist item** | | **Guiding question(s)** | **Location** |  |
| --- | --- | --- | --- | --- |
| 1 | Interviewer/facilitator | *Which author/s conducted the interview or focus group?* | Methods: Data collection | **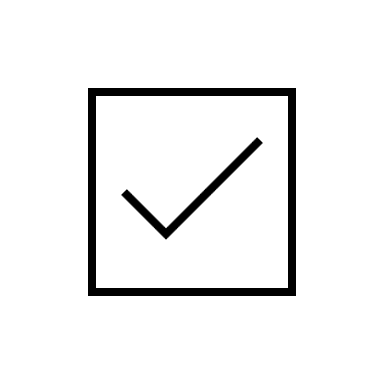** |
| 2 | Credentials | *What were the researcher’s credentials? E.g. PhD, MD* | Title page | **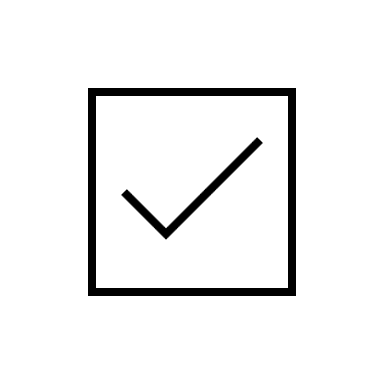** |
| 3 | Occupation | *What was their occupation at the time of the study?* | Methods: Study design | **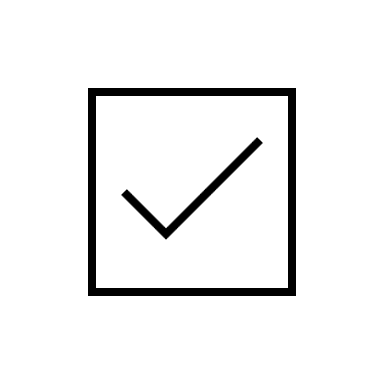** |
| 4 | Gender | *Was the researcher male or female?* | Methods: Study design | **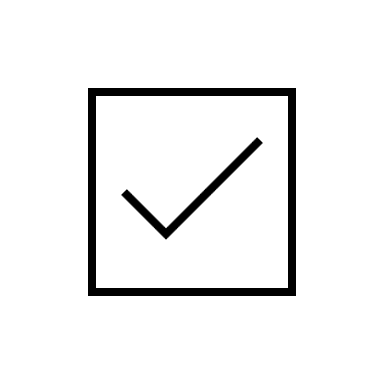** |
| 5 | Experience and training | *What experience or training did the researcher have?* | Methods: Study design | **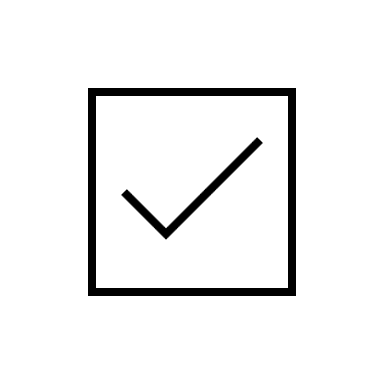** |
| 6 | Relationship established | *Was a relationship established prior to study commencement?* | Methods: Study design | **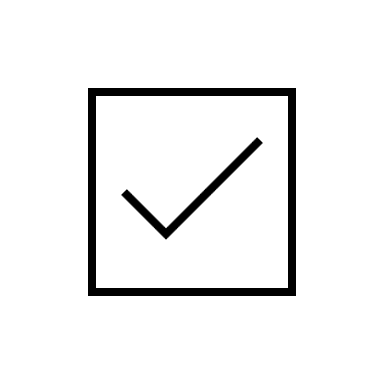** |
| 7 | Participant knowledge of the interviewer | *What did the participants know about the researcher? e.g. personal goals, reasons for doing the research* | Methods: Study design | **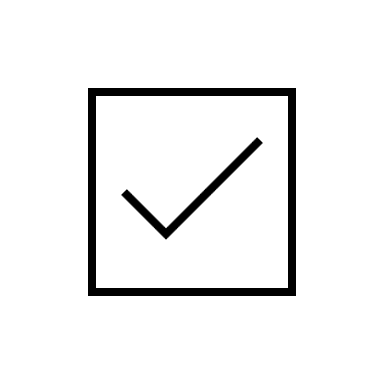** |
| 8 | Interviewer characteristics | *What characteristics were reported about the interviewer/facilitator? e.g. Bias, assumptions, reasons and interests in the research topic* | Methods: Study design | **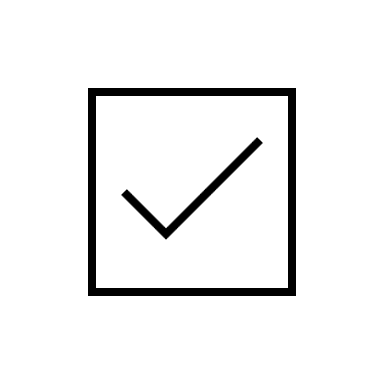** |
| 9 | Methodological orientation and theory | *What methodological orientation was stated to underpin the study? e.g. grounded theory, discourse analysis, ethnography, phenomenology, content analysis* | Methods: Study design | **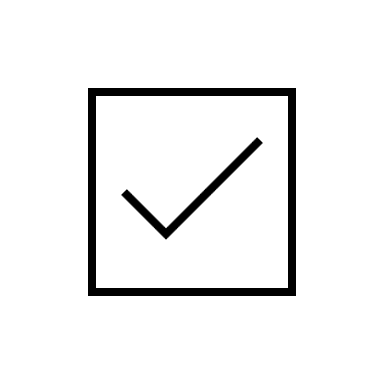** |
| 10 | Sampling | *How were participants selected?* *e.g. purposive, convenience, consecutive, snowball* | Methods: Participant sampling and recruitment | **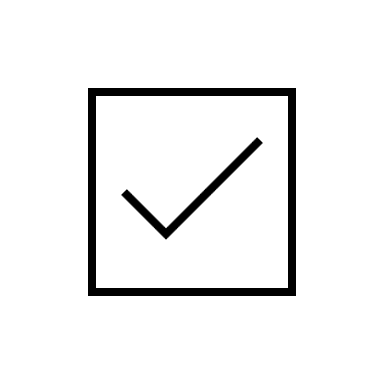** |
| 11 | Method of approach | *How were participants approached? e.g. face-to-face, telephone, mail, email* | Methods: Participant sampling and recruitment | **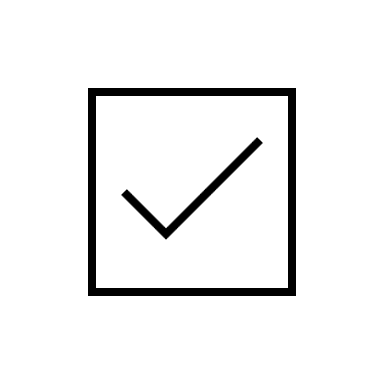** |
| 12 | Sample size | *How many participants were in the study?* | Results | **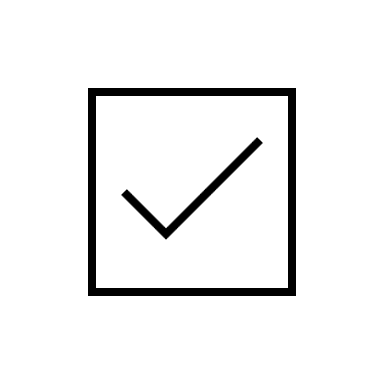** |
| 13 | Non-participation | *How many people refused to participate or dropped out? Reasons?* | Results | **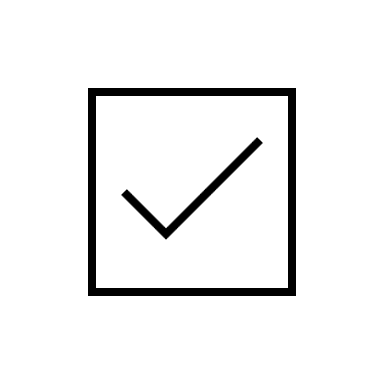** |
| 14 | Setting of data collection | *Where was the data collected? e.g. home, clinic, workplace* | Methods: Data collection | **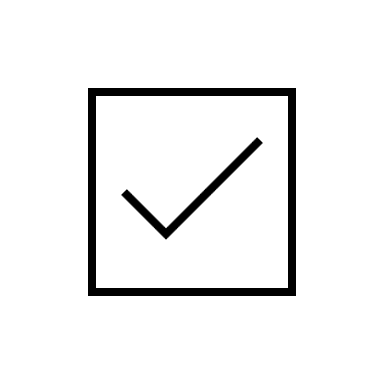** |
| 15 | Presence of non-participants | *Was anyone else present besides the participants and researchers?* | Methods: Data collection | **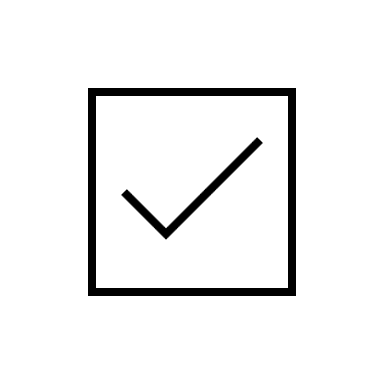** |
| 16 | Description of sample | *What are the important characteristics of the sample? e.g. demographic data, date* | Methods: Participant sampling and recruitment | **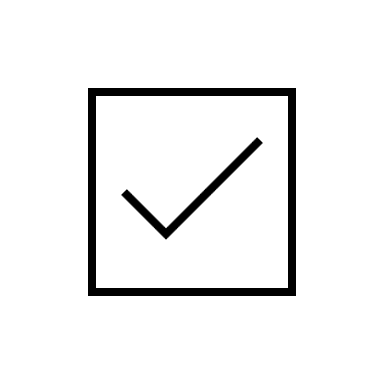** |
| 17 | Interview guide | *Were questions, prompts, guides provided by the authors? Was it pilot tested?* | Methods: Data collection; Supplementary file 3 | **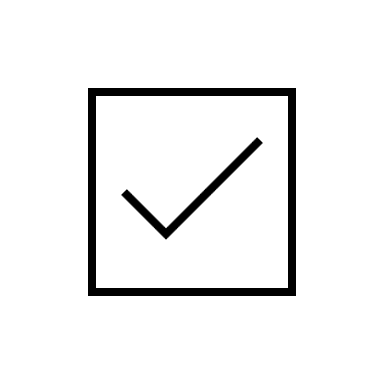** |
| 18 | Repeat interviews | *Were repeat interviews carried out? If yes, how many?* | Methods: Data collection | **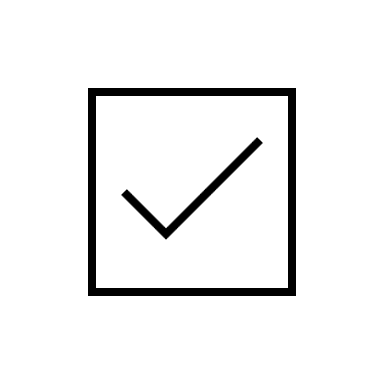** |
| 19 | Audio/visual recording | *Did the research use audio or visual recording to collect the data?* | Methods: Data collection | **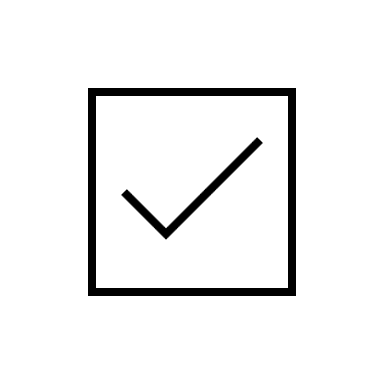** |
| 20 | Field notes | *Were field notes made during and/or after the interview or focus group?* | Methods: Data collection | **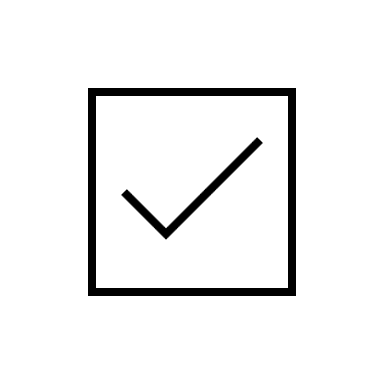** |
| 21 | Duration | *What was the duration of the interviews or focus group?* | Methods: Data collection | **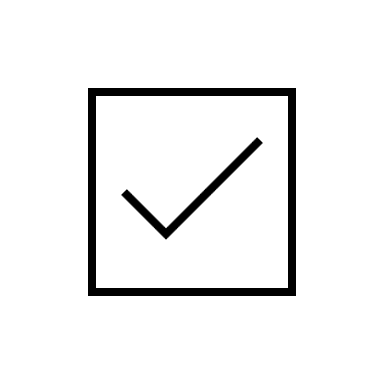** |
| 22 | Data saturation | *Was data saturation discussed?* | Not appropriate for study design.^[[1]](#footnote-1)^ Recruitment endpoint described in Methods: Participant sampling and recruitment | **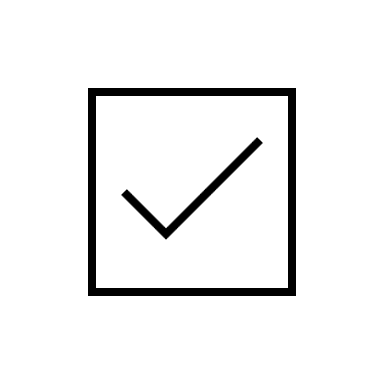** |
| 23 | Transcripts returned | *Were transcripts returned to participants for comment and/or correction?* | Methods: Data collection | **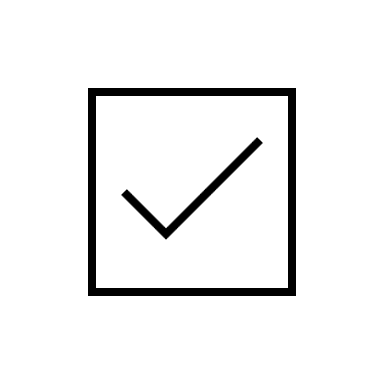** |
| 24 | Number of data coders | *How many data coders coded the data?* | Methods: Data analysis | **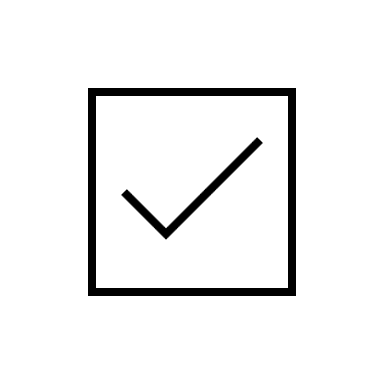** |
| 25 | Description of the coding tree | *Did authors provide a description of the coding tree?* | Methods: Data analysis; Supplementary file 4 | **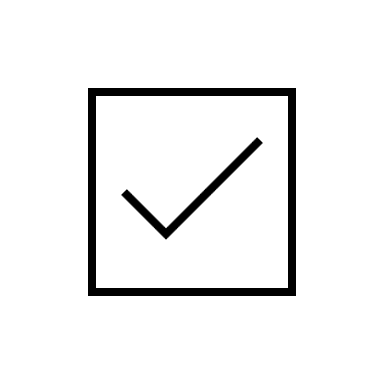** |
| 26 | Derivation of themes | *Were themes identified in advance or derived from the data?* | Methods: Data analysis | **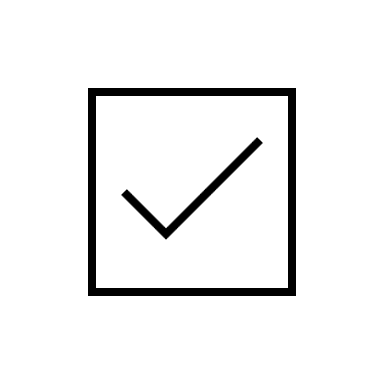** |
| 27 | Software | *What software, if applicable, was used to manage the data?* | Methods: Data analysis | **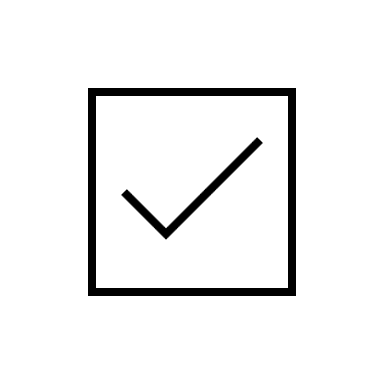** |
| 28 | Participant checking | *Did participants provide feedback on the findings?* | Methods: Data analysis | **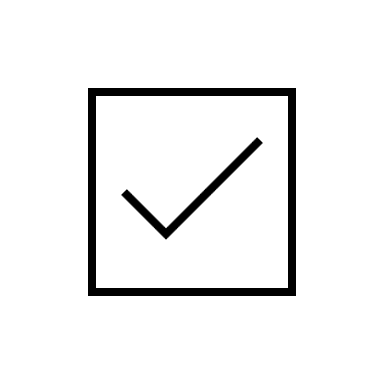** |
| 29 | Quotations presented | *Were participant quotations presented to illustrate the themes / findings? Was each quotation identified? e.g. participant number* | Results | **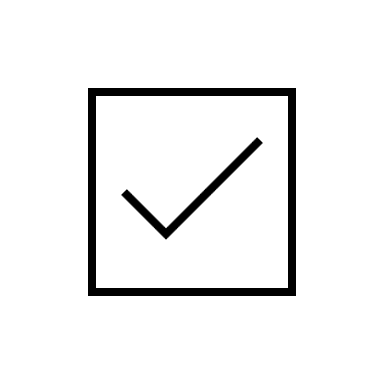** |
| 30 | Data and findings consistent | *Was there consistency between the data presented and the findings?* | Results | **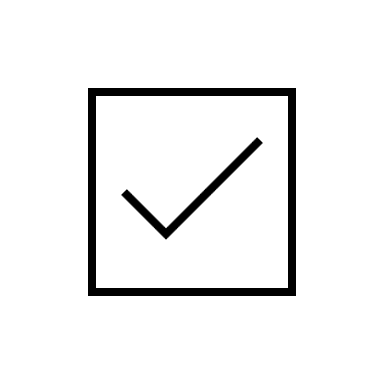** |
| 31 | Clarity of major themes | *Were major themes clearly presented in the findings?* | Results | **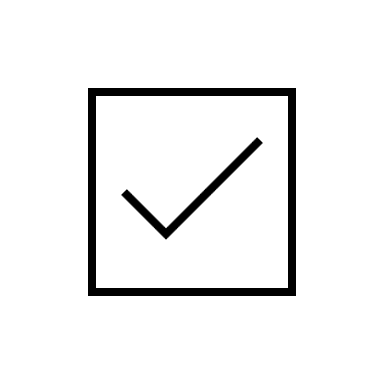** |
| 32 | Clarity of minor themes | *Is there a description of diverse cases or discussion of minor themes?* | Results | **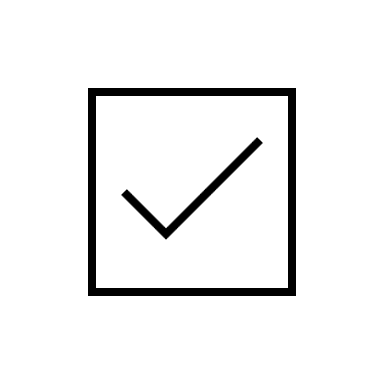** |

* Checklist adapted from Tong A, Sainsbury P, Craig J. Consolidated criteria for reporting qualitative research (COREQ): a 32-item checklist for interviews and focus groups. *Int J Qual Health Care*. 2007 Dec 1; 19 (6): 349-57.

1. For more information on why data saturation is not appropriate for reflexive thematic analysis, see: 1) Braun V and Clarke V. To saturate or not to saturate? Questioning data saturation as a useful concept for thematic analysis and sample-size rationales. Qual Res Sport Exerc Health 2021; 13: 201–216; 2) Braun V, Clarke V. Supporting best practice in reflexive thematic analysis reporting in Palliative Medicine: A review of published research and introduction to the Reflexive Thematic Analysis Reporting Guidelines (RTARG). Palliative medicine. 2024 Jun;38(6):608-16. [↑](#footnote-ref-1)
