# Supplementary material for: Making sense of pediatric death: An exploratory qualitative study of emotion management strategies applied by the pediatric intensive care unit interprofessional team
Source: Palliat Care Soc Pract. 2025 Nov 12;19:26323524251393267. doi: 10.1177/26323524251393267 (PMC12612547; doi:10.1177/26323524251393267)
Supplement: sj-docx-2-pcr-10.1177_26323524251393267 – Supplemental material for Making sense of pediatric death: An exploratory qualitative study of emotion management strategies applied by the pediatric intensive care unit interprofessional team [file sj-docx-2-pcr-10.1177_26323524251393267.docx]

Study Intake Form: Making Sense of Pediatric Death in the PICU

Thank you for your interest in our interview-based study about the emotional experiences of working with critically ill children.

This REDCap study intake form asks you to provide your preferred contact information so that the study team can follow up to arrange an interview at a time that is convenient for you. This interview can take place via phone or video-call, depending on your preference, and will take about 30-60 minutes total.

We are also asking a few demographic questions. This is to ensure our study reflects the diverse range of experiences among the interprofessional PICU team.

We aim to interview up to 15 PICU team members for this study. Please note that not everyone who completes this intake form will be contacted for an interview.

By completing this study intake form, you are agreeing to allow the study team to retain the information you have provided for the duration of the study, and to potentially be contacted by the study team to arrange an interview. Study participation is completely voluntary and you will be provided with additional information if contacted. This study intake form does not replace formal consent to participate in the study.

Thank you!

Have you encountered at least one pediatric death in Yes


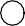

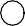


the workplace? No

Name (First and Last):

Preferred method of contact: Email


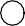

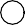

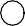


Phone

No preference

Email Address:

Phone Number:

Preferred time of day to be contacted:

Preferred interview setting: Phone


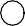

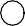

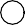


Video-call

No preference

Profession: Chaplain


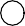

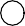

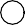

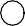

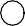

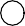

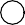

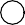

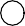


Child life specialist Dietician

Nurse Pharmacist Physician

Respiratory therapist Social worker

Other

Please specify 'other':

Province/territory of current practice: Alberta


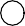

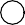

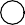

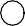

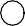

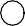

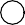

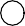

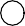

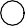

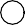

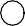

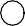


British Columbia Manitoba

New Brunswick Newfoundland and Labrador Northwest Territories

Nova Scotia Nunavut Ontario

Prince Edward Island Quebec Saskatchewan Yukon

How many years have you been employed in your < 1 year


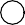

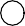

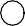

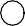

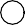


profession? 1-4 years

5-9 years

10-15 years

>15 years

How many years have you worked in the PICU? < 1 1-4


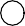

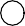

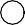

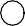

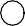


5-9

10-15

>15

Which of the following best describes your current Full-time PICU


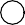

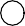

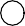


role in the PICU? Part-time PICU

Full-time and I support more than one unit in my role


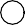
 Part-time and I support more than one unit in my role

Please select the option that best describes your Woman


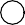

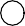

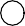

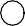


gender: Man

Gender fluid, non-binary, and/or Two-Spirit I prefer not to answer

Do you self-identify as Black, Indigenous, and/or a Yes


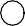

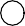

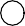


person of colour? No

I prefer not to answer

If there is any other information you believe is important for the study team to know regarding your

potential participation in this study, please indicate that here.

Thank you for completing this study intake form.

If selected for an interview, a study team member (Eva, Molly, Lisa) will get in touch via your preferred method of contact. We plan to contact interviewees starting this summer. The latest you may be contacted is December 2022. If you change your mind about this study, you may request that the study team permanently delete the information you have provided at any time.

If you have any questions about this study intake form or would like more information about the study, please contact Lisa Albrecht at [lalbrecht@cheo.on.ca.](mailto:lalbrecht@cheo.on.ca)

Thank you for your interest in this research.

However, as this study deals with participants' emotional responses to encountering pediatric death in the workplace, you will not be contacted for an interview.

If you would like more information about the study or have any questions about this, please contact Lisa Albrecht at [lalbrecht@cheo.on.ca.](mailto:lalbrecht@cheo.on.ca)
