# Supplementary material for: Making sense of pediatric death: An exploratory qualitative study of emotion management strategies applied by the pediatric intensive care unit interprofessional team
Source: Palliat Care Soc Pract. 2025 Nov 12;19:26323524251393267. doi: 10.1177/26323524251393267 (PMC12612547; doi:10.1177/26323524251393267)
Supplement: sj-docx-3-pcr-10.1177_26323524251393267 – Supplemental material for Making sense of pediatric death: An exploratory qualitative study of emotion management strategies applied by the pediatric intensive care unit interprofessional team [file sj-docx-3-pcr-10.1177_26323524251393267.docx]

**MAKING SENSE OF PEDIATRIC DEATH: Draft interview guide**

**Pre-interview, reiterate the following**:

Thank you again for speaking with me today. We are interviewing you today because we are interested in finding out how people who work in the PICU manage their feelings related to providing end-of-life care. As a reminder, there are no right or wrong answers. We are interested in hearing about your thoughts, feelings, and experiences.

The interview is completely confidential, and you are welcome to skip any question you’d like, pause the interview, or stop it at any time, no questions asked. Should you experience discomfort or distress discussing your experiences with end of life care in your work we can offer you a referral to support resources for support if you wish.

The audio portion of our conversation today will be recorded with your consent, as we discussed earlier, and we will send you a copy of the transcript for you to review. That will give you a chance to make sure we’ve properly cut out any identifying information, as well as give you a chance to make sure you’re happy with what’s in the transcript. If you’d like to further correct, redact of clarify any of the conversation, you are welcome to do so at that point. We want you to be comfortable that the final transcript reflects your thoughts and perspective accurately.

Do you have any questions for me before we begin? [Once all questions are addressed and they are ready to proceed, let them know you will start the recording and begin.]

*Interviewer, please note: Should participant demonstrate signs or cues of emotional distress during the interview, pause the interview, explore the participant’s needs and provide referral to immediate support.*

**A. Background**

1. How long have you been working in the PICU?
2. How would you describe your role when it comes to end of life care in the PICU?

**B. Experiences with death and dying in the PICU**

1. Could you tell me about some of your experiences with death and dying in the PICU?
   1. *Prompt*: What patient deaths stand out for you, if any?

*Probes:* What about this experience made it stand out? Are there any others that come to mind?

1. In your opinion, what makes a PICU death easier or harder to manage?
   1. *Prompts*: What kinds of things impact how easy/hard it is to manage? – For example, is there anything about the family? The trajectory of the child’s illness? How so/In what ways?

**Professional culture and training**

1. What kind of preparation, if any, did you receive about the emotional aspects of encountering death as part of work?
2. *Probe*: What has that emotional impact looked like for you?
3. How would you describe the general attitude toward death in the PICU?
4. *Probe*: How does this compare to your experiences and perspective on death personally? [If they talk about ways their perspective aligns with the PICU environment, can probe further re: any differences or points of tension they have noticed and vice versa].
5. How does the approach to death in the PICU differ from other units, in your opinion?
6. *Prompts*: NICU, palliative care, oncology, adult settings?
7. In your opinion, how does your view on death and dying compare to your colleagues?
8. *Probes:* Are there any differences between professions that you’ve noticed? If so, please tell me about them.

**D. Emotion management**

1. What do you feel the emotional expectations are at work when a patient dies?
   1. *Probe*: What is that like for you?
   2. *Probe*: Where do these assumptions/expectations come from? How do you meet them?
2. Can you think of a time at work when what you felt didn’t match what you thought you ‘should’ feel? If so, what was that like?
3. *Probe*: How often does that happen? What do you do in that situation?

**E. Supports and future training**

1. After a patient dies, what helps you process the experience ?
2. *Prompt*: Any specific people, places, activities, etc.?
3. What do you wish you had known at the start of your career about the emotional aspects of your job?
4. *Prompt*: What would you like a trainee to know now?
5. What supports would you like to see for clinicians who work with critically ill children moving forward?
6. *Prompts*: What would you like PICU administrators to know about the emotional aspects of your job? What kind of structural or institutional supports would you like to see from the PICU or hospital, specifically for folks like you who work with critically ill children?

**F. Wrap up**

1. Sometimes being on the subject brings a lot of things to mind. Is there anything you expected us to ask about that we didn’t get to?
2. Is there anything else we haven’t covered that you would like to add before we wrap up today? [*If no*: Turn off recording + Thank you for sharing your experiences with me today. We really appreciate it.]

**Resources:** Once recording is off, take a moment to check in with the interviewee and remind them of resources. Reiterate that it’s understandable that talking about this may produce an emotional reaction. Should participant demonstrate signs or cues of emotional distress after the interview, explore the participant’s needs and provide referral to immediate support. If they would like additional support, here are several options: they can contact their primary care provider, Employee Assistance Programs (EAP), or one of the many help resources listed in the consent form. Remind them that there is a list of provincial and national crisis and talk lines available in the consent form, for their reference.

- Canada-wide: Crisis Services Canada, 1-833-456-4566 (toll free), http://www.crisisservicescanada.ca
- First Nations and Inuit Hope for Wellness Help Line: 1‑855‑242-3310 (French, English. Cree/Ojibway/Inuktut upon request)
- Newfoundland: Doorways (709) 753-4903; Mobile Crisis Response Team (709) 437-4668; Adult Central Intake (709) 742-8888; bridgethegap.com; and any Hospital Emergency Room
- Nova Scotia: Mental Health Crisis Line, 1.888. 429.8167 (toll free)
- Quebec: Info-Social 811, 811 (no area code), https://www.quebec.ca/en/health/finding-a-resource/info-social-811
- Ottawa, Ontario: Distress centre of Ottawa and region, 613-238-3311, http://www.dcottawa.on.ca
- Toronto, Ontario: Distress centres of Toronto, 416-408-4357, http://www.torontodistresscentre.com
- Hamilton, Ontario: Crisis Outreach and Support Team (COAST) 905-972-8338
- London, Ontario: Reach Out, 519-433-2023
- Manitoba: Klinic crisis line, 1-888-322-3019
- Alberta: Mental Health Help Line, 1-877-303-2642 (toll free)
- British Columbia: 310Mental Health support, 310-6789 (no area code, toll free)
