# Supplementary material for: Making sense of pediatric death: An exploratory qualitative study of emotion management strategies applied by the pediatric intensive care unit interprofessional team
Source: Palliat Care Soc Pract. 2025 Nov 12;19:26323524251393267. doi: 10.1177/26323524251393267 (PMC12612547; doi:10.1177/26323524251393267)
Supplement: sj-docx-4-pcr-10.1177_26323524251393267 – Supplemental material for Making sense of pediatric death: An exploratory qualitative study of emotion management strategies applied by the pediatric intensive care unit interprofessional team [file sj-docx-4-pcr-10.1177_26323524251393267.docx]

Making Sense of Pediatric Death Codebook

Version 5. August 03, 2023

| Code Name | Description |
| --- | --- |
| **Framing the work** | How the interviewee approaches their work at end of life / how they interface with the content of the work – e.g., pharmacist interfaces with work through helping with comfort levels by prepping sedative, physio interfaces differently with child’s comfort (PT might feel more visceral, physical connection, potential that pharm feels more disconnected, work does not involve a physical connection).  Examples: “I’m just there to put out fires”, people who feel they are in more control of the environment, emotional separation from work/when describing tasks  Note: this code will provide context in terms of how they approach the work. Listing what the tasks are = “work context” code. It is possible that the same excerpt may include both! |
| **Death context** | What makes a death “good”/ “bad”/ “normal” from interviewee’s perspective; what deaths are more/less impactful than others for interviewees. Includes examples of cases they have experienced as well as anticipated impacts. |
| **Emotional experience** | Descriptions of emotional experience – e.g., feeling shock/stunned, feeling angry, feeling safe/not safe, etc. Also includes physical effects resulting from emotional experience e.g., getting sweaty |
| **Expectations** | Expressing assumptions or expectations of how things “should” or “would” happen. Includes emotional expectations, expectations of oneself, as well as clinical/performance expectations as they relate to the interviewee’s experiences of encountering death at work. E.g., ‘nobody told me it would be like this; I didn’t expect to feel this sad’ |
| **External influences** | External factors that influence participant’s experience of emotional impacts at work. For example: cultural shift in attitude toward mental health, working during the pandemic, working short-staffed, etc, feeling of public expectations (“I wasn’t on team hero”) |
| **Family outcomes** | Impact of knowing/not knowing how things worked out for the family. Includes feelings around closure, follow-up with families, hearing back from families, ‘the unknown’ when it comes to family outcomes, ‘I don’t want to know what happens’ |
| **Impact on professional self** | What impacts interviewee’s emotional experience of death/dying at work. For example: “Helps when I hear back from someone”, feeling impacted by family’s approach to coping/if family is in denial or acceptance of death |
| **Intersection of work and personal** | How working with death and dying intersects with interviewee’s personal life and personal thoughts on death and dying. For example: Worrying about their own kids, “taking it home”, reflections on own spirituality, “not everyone’s experience at a trampoline park” |
| **Memory** | How remembering (or not remembering) deaths/ details about children who died etc. impacts the interviewee |
| **Navigating emotional tensions** | Experiencing and navigating *internal* tensions. For example: holding two conflicting feelings at the same time; experience of feeling something that they ‘shouldn’t’, or not accessing the emotion they feel they ‘should.’  *Theory to keep in mind for later: core concepts under emotional labour of emotional dissonance, feeling rules* |
| **Navigating ethical tensions** | Experiencing and navigating ethical tensions related to end-of-life care at work. For example: conflict between team and family ideas of best way forward, feeling like they had to pick a side, etc.  *Theory to keep in mind for later: ethical schisms.* |
| **Organizational support** | Both good/bad experiences with organizational/institutional support, things they want from organization, things the hospital can do to improve. Includes CISM training/support, simulations, specific preparation for practice. |
| **Prevented from helping** | Directly prevented by someone from helping in the way they thought best (e.g., family did not want help, tasks delegated to someone else). This code would *not* apply when the person tried to help unsuccessfully, or could not help because they were indirectly prevented (e.g., lack of resources, not enough time) |
| **Qualifiers** | Rationalizations, caveats, anything that softens what they are trying to say or puts them in a less bad light than may be perceived – e.g. “every death is sad, but X is especially sad.” Includes walking back statements to sound less emphatic. |
| **Recognition** | Both feeling recognized and feeling invisible; importance of acknowledgement, validating the impact that they’re having. Includes recognition from families, peers, supervisors. Includes recognition of their skills/feedback/emotional burdens – anytime interviewees mentioned their experience of feeling seen/understood (or not) goes here. |
| **Relationships** | A parent code for all relational phenomena and relationships between the interviewee and how those intersect with their lived experience of encountering death at work. Code at child codes by type of relationship. If there is not an appropriate child code, but it feels relevant to relationships, code at “Relationships” |
| *Team relationships* | Experiences and impacts of informal peer support, sense of community with colleagues. |
| *Mentorship* | The respondent acts as mentor to newer colleagues or is mentored by more senior colleagues. Can include physical, emotional, or strategic components. Specifically addresses colleague at different career point and has teaching component. |
| *Family relationships* | Experience where relationship with family of patient impacted experience with death and dying/managing their emotions in relation to death and dying (e.g., interviewee’s perception on family’s ability to cope).  Note: This is different from the “Family outcomes” code which refers to the objective knowing about the families journey after the death (e.g., being able to see how a family is doing providing closure). |
| *Patient relationships* | Experience where relationship with patient impacted experience with death and dying/managing their emotions in relation to death and dying (e.g., HSC001’s experience when 24y/o patient died). |
| **Helpful connections** | Includes experiences with formal peer support, clinical coaches, social mechanisms as resources. Also include experiences with external training. |
| **Strategies** | Strategies for managing their emotions at work, making sense of death/dying in this context, coping mechanisms. For example: maintaining “neutrality” – supressing feelings, “boxing it up”, putting aside one thing for another; “having no opinion”, “not allowing yourself to judge”; self-silencing; Focus on the family/ focus on supporting the family, needing to slow down/take time, setting boundaries  *Theory to keep in mind for later: core concepts under emotional labour of deep/surface acting* |
| *Compartmentalization* | Sub-code of strategies. All the ‘boxing it up’, creating specific boundaries, etc. excerpts would be coded here as well as at the ‘parent code’ Strategies. Includes helpful and harmful compartmentalization (e.g. ‘it’s not my grief’, ‘I get to go home’) |
| **Influences over time** | Ways in which emotional impacts have evolved/changed over time for the interviewee personally (e.g., over different career stages, over amount of exposure to the environment/context/death at work) |
| **Work context** | Describing their role or specific tasks in end-of-life care |
